# General Overview of Radon Studies in Health Hazard Perspectives

**DOI:** 10.1155/2021/6659795

**Published:** 2021-07-31

**Authors:** Guadie Degu Belete, Yetsedaw Alemu Anteneh

**Affiliations:** ^1^Physics Department, College of Natural and Computational Sciences, Assosa University, Assosa, Ethiopia; ^2^Environmental Science Department, College of Natural and Computational Sciences, Assosa University, Assosa, Ethiopia

## Abstract

The adverse human health effects due to ionizing radiation are well known. Radon is the major source of background radiation among those that are of natural origin. It contributes about 55% of the natural radiation dose to humans. It is a colorless, odorless, and tasteless radioactive noble gas that comes from the natural radioactive decay series of uranium. Radon can be found everywhere in the atmosphere and become attached to aerosols in the air. The aerosols carrying radon and its progeny can be inhaled and deposited in different regions of the human respiratory tract. The deposited radioactive aerosols continue to decay and exposing the lung to ionizing radiation can destroy sensitive cells in the lung, causing a mutation that turns to be cancerous. Different countries and international and national organizations put their action levels to reduce radon lung cancer risk. The Environmental Protection Agency recommends 148 Bq/m^3^ as the action level. On the other hand, International Commission for Radiation Protection (ICRP) recommends 200 Bq/m^3^ as the action level. The main objective of this review is to focus on how radon is established as a health hazard, ways of radon detection and measurements, methods of reducing and controlling high indoor radon concentration, and what are the recommended international action levels of radon concentrations. It mainly focuses on the health perspective of radon studies because it is now a crucial and hot issue in the world. In most developing countries like our country Ethiopia, radon studies are not well investigated.

## 1. Introduction

The discovery of radioactivity left a bright spot on the birth and progress of nuclear investigations. While Becquerel was investigating the possibility that fluorescent materials emit X-rays when stimulated by light, he discovered the radioactivity of uranium in 1896 before the sensitive nuclear investigations were started in 1899. Soon afterward, Pierre and Marie Curie, under extracting uranium from the ore, found two other elements that are also radioactive; the first one is polonium and the second one is radium that is thousand times more radioactive than uranium [1]. Marie and Pierre Curies were the first to observe that the decay product of radium is radioactive. The gaseous state of the product was not known. The work of Ernest Dorn (1900) and Rutherford (1901) confirmed that the emanation from radium was a radioactive gas. Ramsay and Gray in 1908 gave the name niton to this emanation. It was called radon since 1923 [2].

For the first time, radon was suspected to the health hazards due to the high incidence of lung cancers among miners. Miners in Schneeberg in Germany and Jachymov in Czechoslovakia were continuously affected by a respiratory disease. Initially, the ore dust was considered a reason for the high incidence of lung cancer. Early in the 20th century, the malignancy was shown to be primary carcinoma of the lung. Lung cancer was found among many radon miners in Schneeberg in Germany and Jachymov in Czechoslovakia where high levels of radon mines were present, which led to the hypothesis that radon was the cause of lung cancer. So that the first investigations were performed on a high concentration of radon exposures of underground miners in their workplaces.

Radon (Rn) is a decay product of radium (Ra), which is a member of the uranium (U) decay chain. The physical and chemical properties of radon such as colorless, odorless, and tasteless radioactive nature make it difficult to detect without special equipment. Radon has three well-known isotopes, radon (^222^Rn), thoron (^220^Rn), and actinon (^219^Rn), which are found from the decay series of uranium isotopes (^238^U, ^236^U, and ^235^U), respectively [3]. The three isotopes of radon (^222^Rn, ^220^Rn, and ^219^Rn) have a half-life of 3.82 days, 55.8 seconds, and 3.98 seconds, respectively. Radon is among the leading contributors to ionizing radiation and it has been identified as a health hazard for mankind. It is the most leading source of the background radiation dose (55%) received by the environment [4, 5], and it is found in variable concentrations from location to location and even from season to season.

Since radon is an unstable atom when it undergoes radioactive decay, it forms a number of short-lived radioactive decay products (called radon progeny), which include polonium (^218^Po), lead (^214^Pb), bismuth (^214^Bi), and polonium (^214^Po). Alpha, beta, or sometimes gamma radiation is emitted out under each radioactive transformation. The successive radioactive transformation continues up to stable lead (^206^Pb) which is the last element of the decay series. Among these radon daughters, the alpha emitter's polonium (^218^Po) and lead (^214^Pb) contribute to the maximum of the radiation dose (over 90%) from exposure to radon [6]. Radon is an inert gas; therefore, it is a noble gas. It is the last member of the noble gas family. Radon does not react with air, water, and others, but its decay daughters are electrically charged so that they are reactive and they are the cause of radiological health effects to humans.

Radon is a radioactive gas that comes from the natural radioactive decay series of uranium in soil, rock, building materials, groundwater, and mining areas. Confined areas of the house such as basements where the air is not moving freely, some openings, and holes of the homes are also sources of radon [7]. Different building materials such as cement, rock, concrete, marble, paints, and gypsum always contain uranium and radium. Nevertheless, the ground is the major radon source. Up to several thousand Becquerel per liter (Bq/l), radon concentration can be measured in water from drilled wells [4].

Radon reaches the surface of the Earth through emanation and exhalation. The radon atom which escaped from the mineral grain into the pore space undergoes decay within the recoil distance of the grain surface. In common minerals, water, and air, the recoil distance of radon (^222^Rn) is 20–70 nm, 100 nm, and 63 *μ*m, respectively. Radon atoms entering the pore space are then transported by diffusion and advection through this space until they in turn decay or are released into the atmosphere (exhalation). Radon generation and transport in porous materials involve the solid, liquid, and gas phases in the process of emanation, diffusion, advection, absorption in the liquid phase, and adsorption in the solid phase [8]. The amount of radon atoms that is released into rock or soil pore space from a radium-bearing grain is called the emanation coefficient. The grain size and shape predominantly determine the emanation of radon in the soil [9].

Radon decay products in the air become attached to the monodispersed and polydispersed aerosols due to their electrostatics nature [10]. Depending on the aerosol concentration of the surrounding environment, the electrostatic charge of the radon progeny and humidity of the surrounding environment about 80% of the decay products will be attached to the aerosols in the air that we breathe in. It can be inhaled and emit radiation that bombards sensitive tissues in the lung causing DNA damage [7].

The radiological health hazards of radon are not limited to the underground miners only; humans inside the buildings and houses are also exposed to radon and its decay products. The major parts of the Earth's crust consist of uranium; soils and various building materials contain uranium. The radon gas that emanates from building materials and soils can deposit in confined places such as buildings and houses; as a result, room occupants can easily inhale radon and its progeny [11]. Radon in the indoor environment can be accumulated to significant levels. The types of construction materials of the building and the soil composition around the house determine the amount of indoor radon concentration [12]. The emanation and diffusion of radon (^222^Rn) gas that migrates to the house depends on those factors. The air pressure difference between the house and the soil causes the emanated radon gas to move from the soil to the house, that is, from high pressure to low pressure. The exhalation of ^222^Rn is more in permeable soils, such as coarse sand and gravel than through impermeable soils, such as clays. The design, construction, and ventilation of the house are the major factors that determine the amount of indoor radon (^222^Rn) concentration [12, 13]. The soil gas emanations from soils before decaying, off-gassing of waterborne ^222^Rn into indoor air, building materials, and outdoor air are the major sources of radon in homes.  Table 1 gives a summary of the principal dwelling radon concentration results from a number of national and regional surveys carried out in European countries, North American countries, Japan, and Australia [15]. In some surveys, the dwellings were chosen in a random and representative fashion, while in others they were chosen from a specially selected group of dwellings.

In the indoor environment especially confined spaces in houses and other buildings where air exchange is not allowed, radon and its daughters can be deposited to harmful levels. Because of its adverse health effect on human, one needs to take action if the radon concentration exceeds the recommended action levels. With regard to this, according to the Environmental Protection Agency (EPA) report, continuous exposure to 148 Bq/m^3^ radon concentrations from 1000 inhabitants' 13–50 persons will have a chance of inducing lung cancer. On the other hand, the International Commission for Radiation Protection (ICRP) recommends 200 Bq/m^3^ as an action level. So one should take action to reduce its concentration. There are several radon mitigation methods in the indoor environment, including the sealing of cracks in floors and walls and adjusting the design of the building to change the flow of air into the building [16]. Improving the ventilation of the house, sealing of cracks and other openings on the walls, improving the ventilation of the room, installing a radon pump system, opening of windows, doors, and vents of the house (called natural ventilation), and house pressurization using a fan to blow air into the basement are the major principles that should be taken to reduce radon gas concentrations in the breathing zones of occupied buildings, homes, offices, and schools. Improving the ventilation of the indoor environment can reduce the radon concentration up to 50 percent [8].

## 2. Health Effects of Radon

The ionizing radiation emitted out from the inhalation and ingestion of radon undergoes radioactive decay that continuously affects the cells of the sensitive organs like stomach and lung, which can damage the DNA and can cause cancer. Thus, naturally occurring radon in buildings has been identified as a human lung carcinogen and hence the radioactive radon gas is established as the leading cause of lung cancer to humans just after smoking tobacco [17].

Radon (^222^Rn) can exist in the indoor and outdoor environment everywhere in the atmosphere. When a person inhaled radon progeny, a part of them can be deposited in different regions of the human respiratory system. Two of the radon daughters, ^218^Po and ^214^Po, are the major alpha particle emitters that impart their energies to the walls of the respiratory system, which can interact with the sensitive cells in the lung [4, 18].

Based on the results from different experiments performed in laboratory animals and epidemiologic cohort studies on uranium miners, radon is classified as human lung carcinogen. The increase in the risk of lung cancer is due to the alpha particles emitted out from its progeny, which depends on the level of exposure. Radon is a gas, so it can be exhaled immediately after being inhaled, but radon decay products that attach themselves to other molecules in the air and other solid particles in the air such as dust, aerosols, and cigarette particles can be deposited on the walls of different regions of the lung. The deposited radon progeny emits ionizing radiation in the form of alpha particles, which can damage bronchial epithelial cells in the lung that could cause cancer [5]. The risk of lung cancer becomes more dangerous if the person under radon exposure is a smoker. According to the Biological Effects of Ionizing Radiation (BEIR) IV report of the US National Academies of Sciences, under the same radon concentration exposure, smokers have 10 times more chance of developing lung cancer than nonsmokers [19].

Since radon (^222^Rn) is a radioactive noble gas, it is an unstable atom and makes a radioactive decay into a number of radioactive daughters as it is observed in the decay series of radium. Polonium-218 and polonium-214 are major sources of adverse health effects among radon daughters. If radon is inhaled, the decay products, whether attached to aerosol particles or unattached, will largely be deposited on the surface of the respiratory tract. The deposition of radioactive dust or aerosols containing radon and its progeny in the human respiratory system depends on both the biological nature of the person and the behavior and nature of the particle. Small size particles diffuse further and are deposited in the lower regions of the respiratory tract and larger diameter particles of the order of ten to hundreds of micrometer deposit in the upper regions of the human airways [17]. As compared to the large size particles, small size radioactive particles are more radiological dangerous as the larger radioactive particles have a probability to be cleared from the airways through different mechanisms. The deposited radioactive atom, dust, or aerosol undergoes a continuous radioactive transformation up to stable lead (^210^Pb). The energetic alpha radiation with some associated gamma radiation too during the decay affects the lung cells by either creating free radicals or causing DNA breaks or damage, perhaps causing mutations [5]. In fact, the radioactive radon progeny will have a probability to be transported to the other parts of the human body through blood circulation.

In the human body, there is a huge amount of water; radiation like alpha (or any other ionizing radiations) interacts with water. The electron produced by ionization energy loss of radiation has interaction with water molecules.

(1) $$\begin{array}{c} {\text{H}}_{2}\text{O}+e^{-}⟶{\text{H}}_{2}{\text{O}}^{-} \end{array}$$

(2) $$\begin{array}{c} {\text{H}}_{2}\text{O}+{\text{e}}^{-}⟶{\text{H}}_{2}{\text{O}}^{+}+2{\text{e}}^{-} \end{array}$$

(3) $$\begin{array}{c} {\text{H}}_{2}{\text{O}}^{-}⟶\text{H}+{\text{OH}}^{-} \end{array}$$

(4) $$\begin{array}{c} {\text{H}}_{2}{\text{O}}^{+}⟶\text{OH}+{\text{H}}^{+} \end{array}$$

All these reactions are possible, the result of the reaction (H_2_O^+^, H_2_O^−^, and H_2_O_2_) reacts with the body cells in our body. Particularly the poison hydrogen peroxide H_2_O_2_ is more dangerous to body cells chemically, which produces more damage to our body.

Radon is a radioactive gas. Therefore, the human body is exposed to radon and its decay products through inhalation [19]. One single most important mechanism by which the lung is exposed to radon daughters is the deposition of aerosols in the different regions of the human respiratory airways. Deposition of aerosols that are inhaled into the lung can be attached to the radioactive progeny of radon. The inhaled radon undergoes a number of spontaneous transformations into its decay products until it reaches a stable lead in the respiratory tract. A successive radioactive transformation is taking place under the decay from one daughter to another. Under each number of nuclear transformations, ionizing radiation is emitted, which contributes to the calculation of the cellular dose of the lung [20]. The deposition of alpha energy in the lungs increases proportionally with increasing radon exposure and radon concentrations. This also results in the increase of the radon lung cancer risk. The biological nature of the reference individual such as lung morphology, breathing pattern, and physical parameters such as fluid dynamics, size, shape, density, charge, and surface properties of the particles of aerosols affects the deposition patterns of aerosols in the human respiratory system.

The risk of developing lung cancer resulting from the inhalation of aerosols carrying radon and its decay products depends on the amount of cellular dose delivered to the sensitive cells of the bronchial epithelium [21]. The dose from radon progeny in the regions of the lung (bronchial (BB), bronchiolar (bb), and alveolar intestinal (AI)) depends on the combined effects of the deposition and clearance of the short-lived radon progeny [22]. This is because all inhaled aerosols will not be deposited; once aerosols have been deposited, clearance begins by several different processes. Following the deposition of radionuclide aerosols in different regions of the respiratory system, clearance occurs in different mechanisms such as through sneezing, coughing, absorption to blood (for soluble particles), and particle transport largely by mucociliary clearance (for insoluble particles).

Different studies have been performed to determine the cellular doses in the human lung resulting from the inhalation of short-lived radon decay products. Since the dose on lung tissue cannot be measured directly, many lung models were developed to deal with the determination of dose delivered by alpha particles in the lung [1]. There is a slight difference among these models in considering airway structure and lung volume. There are three lung models. These are RADEP/IMBA, RADOS, and IDEAL lung models. This difference among models is due to their assumptions that are their lung morphology (lung structure or volume) assumptions. In the RADEP/IMPA model, the bronchial region of the lung has been divided into two compartments, the bronchial (BB) and bronchiolar (bb) region; in the RADOS model, the bronchial tree is composed of symmetric airway generations; and in the IDEAL model, the bronchial region is considered to consist of 12 to 20 asymmetric airway generations. Due to these morphometric differences, the deposition, clearance, and cellular doses have significant differences among models. Due to the morphometric differences among models, the calculation of deposition, clearance, and cellular doses are different from model to model. In RADEP/IMPA model, these calculations are determined by considering regions, whereas in the case of generation models (RADOS and IDEAL models), these calculations are determined by considering airway generations or individual airways [23]. The human respiratory system is divided into five main regions based on differences in radiosensitivity, deposition, and clearance according to HRTM. The regions are the extrathoracic (ET) region, bronchial (BB) region, bronchiolar (bb) region, and alveolar interstitial (AI) region that is called the gas exchange region. The extrathoracic region that is called the head and neck region of the respiratory system is divided into ET1, which includes the anterior nasal passage, and ET2, which consists of the posterior nasal and oral passages, the pharynx, and larynx. The bronchial region of the human lung consists of a sequence of bifurcating tubes, which decrease in diameter and length as they penetrate deeper into the lung until the terminal bronchioles are reached. All airways beyond the terminal bronchioles are surrounded by alveoli [21]. Generally, the radon progeny which can be inhaled through nose or mouth passes from the pharynx and larynx to trachea which has zero generation (has no airways) and then enters into the bronchial regions which consist of 0–17 airway generations and finally passes into a gas exchange area (alveolar region) which starts from 17 to 23 generations.

The amount of energy associated with the emission of alpha radiation from the decay chain of radon until it becomes a stable lead (^206^Pb), which bombards the human tissue, is called potential alpha energy. The absorbed dose reflects the energy deposited per unit mass, not the total energy. Radon dose *D* is the amount of energy delivered from radon and its daughters per unit exposure mass, for example, per unit human body and per unit human lung. It measures the average density of energy absorbed by the mass of the absorbing tissue. Its SI unit is J/Kg. The special name given for this unit is Gray (Gy). Dose equivalent H is the product of the absorbed dose *D* and *W*_*R*_ is called the radiation weight factor. It is the quantity that measures both the amount of energy absorbed by the target mass and also the biological effectiveness of the radiation.

(5) $$\begin{array}{c} H=D\times W_{R}. \end{array}$$

It is the quantity that measures the risk of that typical radiation. The risk on the tissue also depends on the tissue type. ICRP introduces the idea of effective dose *E*. The effective dose of a particular tissue can be calculated by multiplying the equivalent dose of that particular body tissue by their respective tissue weighting factor of that tissue.

(6) $$\begin{array}{c} E=H\times W_{T}. \end{array}$$

In the determination of the effective dose in the human lung resulting from inhalation of short-lived radon progeny, we need to consider the tissue weight factor for the lung, which indicates the sensitivity of the target tissue for the incoming radiation. Radiation damage in biological tissue depends not only on the kind of radiation but also on the kind of tissue. Different organs or parts of the human body show different sensitivities to radiation exposure; different tissue has different sensitivity for radiations. Most of the dose from radon progeny is due to irradiation from alpha particles, the radiation weight factor of alpha particle *W*_*R*_ is 20, and the tissue weight factor of the lung *W*_*T*_ is 0.12 [24].

## 3. Radon Safe Limits

Radon safe limits vary in different regulating agencies and organizations. The Miners Safety and Health Act (MSHA) covers underground miners, whereas the Occupational Safety and Health Act (OSHA) regulates exposure to ^222^Rn gas and ^222^Rn progeny for workers other than miners. The MSHA states that workers should not be exposed to radon and radon progeny in their workplaces if the concentration exceeds 1.0 WL or 100 pCi/L as the action level. It puts the annual action level to be less than 4 WLM per year. OSHA limits exposure to either 30 pCi/L or 0.33 WL based on continuous workplace exposure for 40 hours per week, 52 weeks per year [25]. In Publication 103, the International Commission on Radiological Protection (ICRP) considered that the internationally established value of 1000 Bq/m^3^ or 27 pCi/L might be used globally in the interest of international harmonization of occupational safety standards [26].

The Environmental Protection Agency (EPA) recommends 4 pCi/L as an action level for radon gas concentration and the homeowners need to take different radon mitigation methods if radon concentration exceeds this value. On the other hand, the World Health Organization (WHO) now establishes 2.7 pCi/L as a radon action level [26]. The high concentrations of indoor radon can be controlled and reduced up to the standard international proposed safe limit of indoor radon by means of radon prevention and mitigation as described in unit one.

Based on the results of different studies on the adverse health effects of radon and its decay products, many countries are forced to establish their own action level for indoor radon to reduce radon lung cancer risk. As a health concern like indoor environment, radon concentration at a workplace should be given similar attention where it lagged behind [26]. Different countries put different action levels at dwellings; Table 2 shows the recommended action levels of some countries for radon in indoor air. Different countries have different action levels to take actions if the radon concentration is above the recommended action levels.

## 4. Radon Measurement Methods

For the measurements of the concentration of radon and its daughters in the air, different techniques have been used. Alpha radiation, beta radiation, and gamma radiation are emitted out under the radioactive decay chain of radon. Therefore, numerous techniques have been developed for measuring these radionuclides based on detecting alpha particles, beta particles, or gamma rays, independently or in some combination. Radon measurement techniques are generally classified as *passive* and *active* methods. In the passive methods of measuring radon, the measurement is usually made over a long period of time and the result is reported as the average over the measured time interval. In the active measurement method, the radon concentration is measured at a given measuring point in time.

A small piece of plastic or film is the basic component of solid-state nuclear track detector (SSNTD). When this plastic detector is exposed to radon, the particles that are emitted out during the radioactive decay chain of radon continuously strike the detector which causes a number of submicroscopic tracks to be produced on the plastic or film. After the exposure of the detector for the required amount of time, the detector is returned to the laboratory for counting the number of tracks on the film. To count those submicroscopic tracks easily, the plastic detector is then allowed to immerse in a caustic solution. The amount of radon concentration of the study environment has a direct relation to the number of tracts recorded on the detector. The difference between the number of tracts per unit area of the detector and the background radiation of the laboratory is proportional to the radon concentration [27].

The detection material and detection chamber are the components of a solid-state nuclear track detector. Most solid-state nuclear track detector uses CR-39 as a detection material member. CR-39 is selected because of its good sensitivity, stability against various environmental factors, and high degree of optical clarity. The detector chamber is a cylindrical cup. Carbon is impregnated in the wall material, polypropylene, to enhance electrical conductivity and to avoid the problem of electrostatic charge. Radon enters the holder with a half-time for entry of about 1 minute, which is short compared with the radon half-life of 3.82 days. This means that the radon concentration inside the detector chamber quickly approaches that of outside. It can be shown that the long-term average radon concentration inside the detector chamber is the same as that outside, despite any variations in the outside concentration. But the radon concentration may be overestimated because the short half-time for entry will allow some thoron to enter the detector [27, 28].

The efficiency of a solid-state nuclear track detector depends on the quality of various etching parameters. CR-39 samples are irradiated using an alpha source. Irradiated CR-39 samples can be etched in a solution [29]. NaOH solution is mostly used as an etchant and has been extensively studied. Varying concentrations of NaOH solutions can be used at different temperatures and periods. An optical microscope with an appropriate magnification can be used to observe the etched tracks. A calibration experiment can be carried out to evaluate the relationship between the track density recorded and the radon concentration [28].

## 5. Conclusions

Our world is full of radioactivity from many sources and living things have been exposed to natural radiation since the existence of life in the universe. Radiation is present everywhere upon which almost half of the natural radiation in which human beings are exposed is caused by radon. It is the second leading cause of human lung cancer. Different studies have been conducted since its discovery in 1923 in relation to its respiratory health hazards in Schneeberg (in Germany) and Czechoslovakia among miners. Different countries and organizations put an action level to reduce radon health risk, but the radon problem must be a global issue. It is clear that radon is found elsewhere around us and its concentration varies from place to place and from season to season; its concentration in the living environment should be measured. Indoor radon is most preferable to be studied than occupational radon studies since it is a place where we spent about 80% of our time. It is possible to take a simple inexpensive radon test and focus on the appropriate radon mitigation techniques.

## Figures and Tables

**Table 1:** Summary of radon surveys in dwellings [14].

Country	Indoor radon concentration (Bq/m^3^)		
	Arithmetic mean	Geometric mean	Geometric standard deviation
Australia	11	8	2.1
Belgium	48	38	2.0
Canada	28	11	3.9
Czech Republic	140	44	2.1
Denmark	59	39	2.2
Finland	120	84	2.1
France	89	53	2.0
Germany	49	37	2.0
Greece	55	44	2.4
Ireland	89	57	2.4
Italy	70	52	2.1
Portugal	62	45	2.2
Mexico	140	90	NA
Netherlands	23	18	1.6
Spain	90	46	2.9
United Kingdom	20	14	3.2
USA	46	25	3.1
Switzerland	78	51	1.8
Republic of Korea	53	43	1.8
Japan	16	13	1.8

^*∗*^Not available at the moment.

**Table 2:** Domestic radon concentrations and action levels in different countries [26].

No.	Country	Average radon concentration in homes (Bq/m^3^)	Action level (Bq/m^3^)	Year set
1	Austria	^*∗*^	200	1990
2	Finland	123	400	1992
3	Belgium	^*∗*^	400	1995
4	China	^*∗*^	100–200	—
5	Greece	^*∗*^	200–400	—
6	Germany	50	250	1988
7	Ireland	60	200	1991
8	Israel	^*∗*^	200	—
9	Lithuania	37	100	—
10	Luxembourg	^*∗*^	250	1992
11	Norway	51–60	200	1990
12	Poland	^*∗*^	400	1994
13	Ireland	^*∗*^	200	1991
14	Sweden	108	400	1994
15	Switzerland	70	1000	1994
16	United Kingdom	20	200	1990
17	European community	^*∗*^	400	—
18	USA	46	150	1994
19	Canada	^*∗*^	800	1988

^*∗*^Not available at the moment.
